# Organoids-on-Chips Technology: Unveiling New Perspectives in Rare-Disease Research

**DOI:** 10.3390/ijms26094367

**Published:** 2025-05-04

**Authors:** Xiangyang Li, Hui Wang, Xiaoyan You, Guoping Zhao

**Affiliations:** 1 Henan Engineering Research Center of Food Microbiology, College of Food and Bioengineering, Henan University of Science and Technology, Luoyang 471023, China; xiangyang_224@163.com; 2 Master Lab for Innovative Application of Nature Products, National Center of Technology Innovation for Synthetic Biology, Tianjin Institute of Industrial Biotechnology, Chinese Academy of Sciences (CAS), Tianjin 300308, China; wanghuih@tju.edu.cn; 3 Haile Laboratory of Synthetic Biology, Tianjin 300308, China; 4 School of Life Sciences, Faculty of Medicine, Tianjin University and Tianjin Key Laboratory of Function and Application of Biological Macromolecular Structures, Tianjin 300072, China; 5 CAS-Key Laboratory of Synthetic Biology, CAS Center for Excellence in Molecular Plant Sciences, Shanghai Institute of Plant Physiology and Ecology, Chinese Academy of Sciences, Shanghai 200032, China; 6 CAS Key Laboratory of Quantitative Engineering Biology, Shenzhen Institute of Synthetic Biology, Shenzhen Institute of Advanced Technology, Chinese Academy of Sciences, Shenzhen 518055, China; 7 Engineering Laboratory for Nutrition, Shanghai Institute of Nutrition and Health, Chinese Academy of Sciences, Shanghai 200031, China

**Keywords:** organoids-on-chips, organ chips, organoids, rare disease

## Abstract

The scarcity of robust models and therapeutic options for rare diseases continues to hamper their preclinical investigation. Traditional animal models and two-dimensional cell cultures are limited in their ability to replicate human heredity-associated traits and complex pathological features. Organoids-on-a-chip approaches open up new frontiers in rare-disease research via the integration of organ chips and organoid technology. This integrative strategy offers immense opportunities for the mimicry of disease-related traits, the clarification of the mechanisms underlying disease, and the prediction of treatment responses in a highly human-related manner. This forward-looking perspective suggests organoids on chips are transformative tools for parsing rare-disease pathogenesis, accelerating therapeutic discovery, and bridging the gap between basic research and precision medicine.

## 1. Introduction

Rare diseases, also referred to as orphan diseases, are characterized by their low incidence and prevalence rates. More than 7000 rare diseases have been identified across the globe according to the World Health Organization, collectively impacting approximately 560 million people [1]. Among these diseases, roughly 80% are hereditary, and half typically develop during childhood. Despite increasing recognition of these diseases, only ~700 drugs addressing 6% of rare diseases have received approval for the treatment of these diseases globally, while effective treatment options for the remaining 94% remain lacking [2].

The dearth of reliable therapies for most rare diseases underscores the need to identify more effective approaches to preventing, diagnosing, and treating these diseases and establish comprehensive rare-disease management strategies that can afford patients a better quality of life and health security. Our etiological understanding of rare diseases often relies on the use of immortalized cell lines and animal models. Undeniably, these conventional preclinical models provide humans with an effective means of exploring the development of some diseases. Animal models, however, fail to adequately recapitulate the specific features of human diseases owing to species differences, leading to the attrition of many candidate drugs during the development process [3,4]. The limited genetic diversity of and pharmacogenomic differences between these animals and humans have contributed significantly to drug failures in Phase I and Phase II clinical trials [5]. Two-dimensional (2D) models lack in vivo-like microenvironments (e.g., multicellular components, dynamic flow, and organ–organ interactions) and cannot truly simulate complex 3D tissue microenvironments or crosstalk between cells and the surrounding matrix, which makes it difficult to reflect the physiological properties of human tissues and organs [6,7,8]. As most rare diseases are heritable and characterized by a complex disease pathology, the extant 2D culture systems and animal models cannot effectively mimic these diseases, impeding clinical research and innovative therapies. These limitations have led to efforts to design 3D culture systems that can be used to more effectively study rare-disease drug responses in vitro. In light of these advances, as of 2022, the U.S. Food and Drug Administration (FDA) announced a major shift in regulatory practices by indicating that animal testing would no longer be required prior to clinical trials [9].

**Figure 1:**
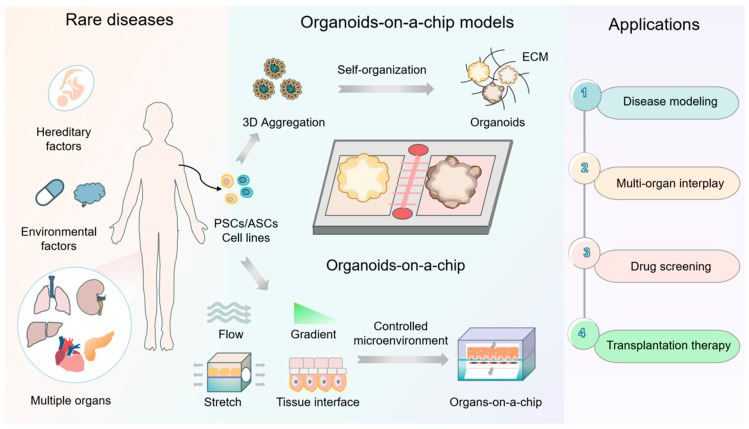
Schematic overview of the existing in vitro organoid-on-a-chip-model rare-disease studies. Rare diseases often result from genetic factors, such as single-gene disorders or chromosomal abnormalities, and environmental exposure such as radiation or chemical exposure. Organoid-on-a-chip systems can create in vivo-like organ equivalents by integrating 3D organoids, biofluids, tissue interfaces, and mechanical stimuli in a biomimetic and controlled manner. They can thus serve as robust platforms for myriad facets of rare-disease research, including disease modeling, multi-organ interactions, drug candidate screening, and studies on transplantation therapy.

Recent efforts to incorporate advanced engineering technologies into the field of developmental biology have fueled the development of new in vitro physiological and pathological models. Organ chips can simulate in vivo physiological conditions, including 3D microenvironments, dynamic processes, and tissue-specific responses [10,11,12]. These chips can also be leveraged for the multifaceted exploration of rare-disease pathologies through the integration of multiple cell types and technologies (e.g., microimaging, sensors, etc.). The importance of this approach is further underscored by the fact that many rare diseases arise due to genetic mutations present during embryogenesis, making efforts to understand the process of organ development valuable. Organoids are characterized by a capacity for self-organization and can reflect the natural cellular arrangements and differentiation activity that arise during organ development, providing an opportunity to investigate the mechanistic basis for myriad rare diseases. As they are composed of multiple cellular components, organoids can effectively recapitulate the genetic heterogeneity and cell–cell interactions that arise in patients [13,14,15]. In April 2025, the FDA issued a historic statement proposing a plan to phase out traditional animal experiments in favor of using laboratory-cultured organoids and organ chip systems to test drug safety. Therefore, the combination of organoid and organ chip technologies provides a powerful biomimetic platform for precise rare-disease modeling and research with the aim of recapitulating multiple pathological manifestations of rare diseases in vitro through the controlled integration of multiple microenvironmental factors, providing new opportunities for the diagnosis and treatment of rare diseases (Figure 1). In this perspective, we offer an overview of the current landscape for organoid-on-a-chip models, highlighting their notable features and pioneering applications for rare-disease research. We also discuss the remaining challenges and summarize the prospects of organoids-on-chips approaches for rare-disease research. In addition, we have provided a table summarizing key information about organoids-on-chips-technology-assisted rare-disease research (Table 1).

## 2. Challenges and the Need for Preclinical Models

The treatment of rare diseases, including rare tumors and rare congenital, immune, neurological, and metabolic disorders, is hampered by difficulties in diagnosis, limited treatment options, and a lack of effective medications [16]. These conditions are characterized by complex etiologies involving genetic mutations, dysregulated signaling pathways, inflammatory activities, and immunological abnormalities, which collectively contribute to disease-related phenotypes. Intra-disease heterogeneity further complicates research and treatment efforts. For example, in regard to Duchenne muscular dystrophy (DMD), patients with the same dystrophic gene mutation may exhibit different clinical trajectories due to modifier genes or epigenetic factors [17]. However, traditional research paradigms struggle to capture the fine micro-diversity of rare diseases, hindering basic research and drug development. Clinical trials for rare diseases are often conducted based on the same structure as phase III clinical trials implemented for more prevalent disorders, but the inherent scarcity of these diseases limits the availability of samples, leading to many barriers to research. The recruitment period for rare-disease trials is three to five times longer than that for common diseases, and these trials are often terminated early due to insufficient sample sizes [18]. Currently, fewer than 200 rare diseases are undergoing Phase III clinical development, underscoring the significant barriers to treatment that most patients with rare diseases continue to face. The heterogeneity of clinical presentations among individuals with rare diseases also makes recruiting patients for Phase I studies difficult, contributing to high frequencies of trial interruption. Most importantly, caution is warranted when interpreting outcomes from successfully executed clinical trials focused on rare diseases, as the diverse manifestations associated with these diseases necessitate consideration of clinical presentation, disease history, and other patient-specific factors. Given the lack of reliable models and the difficulties associated with conducting clinical trials for rare diseases, there is an urgent need to develop alternative in vitro models that can overcome the current barriers. These models should be capable of capturing the complexity and heterogeneity of rare diseases, providing more accurate representations of disease mechanisms and patient variability. This will facilitate the identification of new therapeutic targets, accelerate the drug-screening process, and improve the success rates of clinical trials. Ultimately, the aim is to advance personalized medical approaches to rare diseases.

## 3. Organoid and Organ Chip Models: An Overview

**Figure 2:**
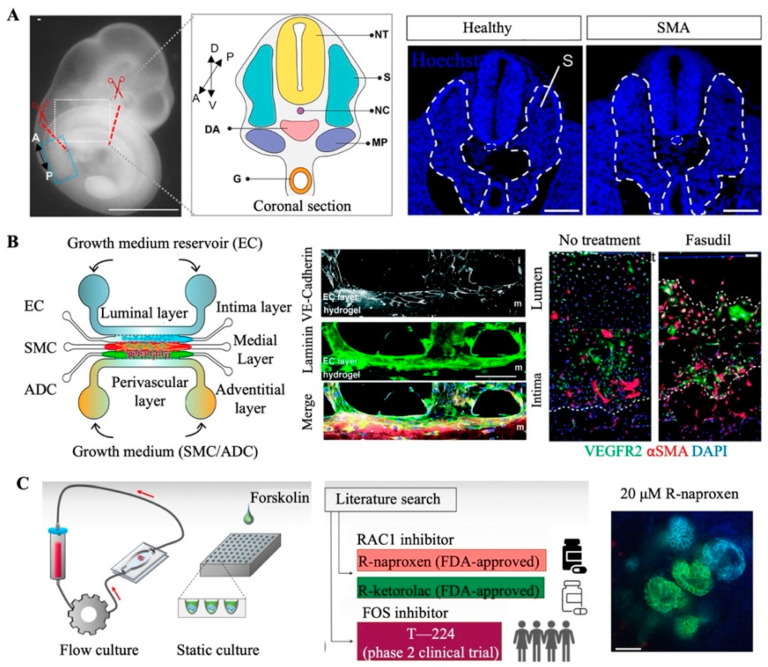
In vitro human-derived organ models for rare-disease research. (**A**) Isogenic patient-derived organoids were used to identify early neurodevelopmental defects linked to the onset of spinal muscular atrophy [20]. This material was reprinted with permission from Grass et al. (Copyright 2024 Elsevier Inc.). (**B**) The use of an organ chip to recapitulate the pathological characteristics associated with pulmonary arterial hypertension [28]. This material was reprinted with permission from Al-Hilal et al. (Copyright 2020 The Royal Society of Chemistry). (**C**) Organoid-on-a-chip model highlighting the pathological mechanosensing mechanisms in the context of autosomal recessive polycystic kidney disease [31]. This material was reprinted with permission from Hiratsuka et al. (Copyright 2022 American Association for the Advancement of Science).

Organoids are 3D, multicellular, self-assembling spheroids derived from various types of stem cells, including pluripotent stem cells (iPSCs), embryonic stem cells (ESCs), and tissue-specific stem cells, retaining the characteristic features of the corresponding organs [15,19]. They can model complex human physiology more accurately than conventional 2D and animal models. Many rare diseases stemming from genetic mutations manifest in the form of abnormal development during childhood, but access to the fetal tissue samples necessary for studying these diseases is extremely limited. Stem-cell-derived organoids represent an attractive alternative for modeling developmental processes, as they consist of various cell types present in early-stage organs. Also, in vitro organoid models derived from patients further facilitate human neurodevelopmental and disease research. For instance, spinal muscular atrophy (SMA) is a neuromuscular disease caused by the loss of spinal motor neurons and muscle atrophy due to decreased levels of motor neuron protein. Grass et al. successfully replicated the early features of this disease, including motor neuron defects and aberrant neural stem cell differentiation, by constructing SMA-patient-derived organoids, allowing researchers to better understand the progression of this neurodegenerative disease [20] (Figure 2A). Additionally, organoids derived from amniotic fluid samples can similarly mirror the morphology and functionality of fetal tissues. They can be used for research focused on fetal development and congenital diseases, including congenital diaphragmatic hernias [21]. Moreover, patient-derived tumor organoids (PDOs) can maintain the distinctive genetic and histological features of the original tumor tissues [22,23]. For example, in rare malignancies such as malignant peritoneal mesothelioma, PDOs can faithfully recapitulate tumor histopathology and genomic heterogeneity, enabling personalized drug testing and expanding the therapeutic options for rare cancers [24]. In a study on inherited kidney diseases, Dvela-Levitt et al. constructed an effective patient-derived model system by using patient-derived cells and pluripotent-stem-cell-derived renal organoids to recapitulate the cellular accumulation of toxic proteins in autosomal-dominant renal tubulointerstitial nephropathy [25]. In summary, organoids are ideal tools for exploring the development and treatment of rare diseases by more closely reproducing the physiological characteristics of human tissues, overcoming the limitations associated with traditional models and advancing our understanding of rare diseases.

Rare-disease development and progression are frequently driven by a confluence of genetic, metabolic, immunological, and/or environmental factors and associated in vivo regulatory processes, including transcription factor activity, intercellular communication, and blood flow. However, the construction of physiologically relevant in vitro models for rare diseases that incorporate multiple cell types, dynamic cell–cell interactions, and extracellular matrices is a challenge [26,27]. Despite the significant advantages of organoids, their immaturity, uncontrollability, and high variability limit their wider application. Designed to address this issue, organ chip technology can independently control or tightly couple various factors, such as mechanical stimuli, fluid dynamics, oxygen gradients, 3D architecture, and compartmentalized spaces, to replicate the native microenvironmental conditions present in human organs [11]. The rare disease pulmonary arterial hypertension (PAH) is closely related to abnormalities in blood flow shear stress and endothelial cell interactions, in which the patient’s pulmonary arteries/arterioles become stiff and occluded, placing a huge burden on the heart or even leading to right heart failure. Al-Hilal et al. successfully modeled the pathophysiology of PAH based on microfluidic chips, in which shear stress induces endothelial thickening, muscle hyperplasia, endothelial mesenchymal transition, and arterial remodeling similar to the elevated mean pulmonary arterial pressure observed in the clinical manifestations of human PAH [28] (Figure 2B). Naik et al. similarly combined 3D proximal tubule chips with fluid shear stress to replicate the features of Lowe syndrome and Dent disease type II. The model of *OCRL* gene deficiency recreated the interstitial fibrosis, reflecting the disease pathology [29]. Rumsey et al. separately utilized hiPSC-derived neurons and Schwann cells to establish organ chips capable of simulating two rare forms of autoimmune neuropathy: chronic inflammatory demyelinating polyneuropathy and multifocal motor neuropathy [30]. These systems recapitulate autoantibody-mediated myelin sheath injuries and reductions in neuroelectric conduction. Moreover, monoclonal antibody inhibitors targeting the complement system have rescued complement-mediated dysfunction associated with disease onset, thereby restoring neurological function. Following their promising results, this team obtained the first FDA investigational-new-drug approval for a clinical trial, relying solely on preclinical efficacy data from human organ-chip studies. In conclusion, organ chip platforms represent a powerful and innovative approach to effectively replicating the pathogenesis of rare diseases and capturing their complex features. Although this technology has been scarcely studied and has low success rates in the field of rare-disease research, it is more accurate and controllable than traditional disease models and opens new avenues for the development of targeted therapies and personalized medicine.

## 4. Development of Organoid-on-a-Chip Models for Rare-Disease Modeling, Drug Development, and Transplantation Therapy

While there have been remarkable advances in the use of organoids and organ chip technologies for rare-disease research, the development of effective therapeutics remains a formidable challenge. The development of organoids relies on leveraging the precise timing and regulation of signaling pathways to guide cellular differentiation and spatial organization [32]. Conventional organoid culture systems, however, lack the ability to precisely control biophysical and biochemical cues during the process of stem cell differentiation. In contrast, organoid chips excellently combine the advantages of organoid and organ-on-chip technologies, integrating the principles of stem cell development and engineering to create a microenvironment in vitro that resembles a given in vivo environment [33,34]. In this system, multiple cell types coexist and collaborate with each other, mimicking the complexity of real tissues. The biofluids in the microchannels not only provide nutrients and allow oxygen exchange for the cells but also mimic the transport of substances and the elimination of metabolic waste in the body [33,35]. In addition, mechanical stimuli such as tensile, compressive, and shear stresses are applied in this system to simulate the various mechanical effects that tissues are subjected to while physiological activities are carried out in the human body [36,37]. Importantly, rare diseases often involve abnormalities in specific tissue layers, and the geometry of microchannels in organoid chips has a significant impact on cell differentiation and the activation of specific pathways [38]. By directing the migration and distribution of cells along specific pathways, microchannel geometry affects tissue formation and structures. It also modifies the local hydrodynamic environment, which in turn modulates the microenvironmental signals to which the cells are exposed, influencing the direction of cell differentiation and the activation of specific signaling pathways. Taken together, these properties make organoid chips suitable for rare-disease research.

By applying dynamic fluid flow, organoid-on-a-chip systems are also able to simulate the pathological conditions associated with rare diseases [10,39]. For example, the formation of renal cysts and the loss of renal function as a consequence of organ enlargement are both observed in autosomal recessive polycystic kidney disease. This rare disease involves urinary-flow-associated cell surface mutations that are not readily observable in static organ systems. To overcome this issue, Hiratsukabo et al. utilized a 3D-printed perfusion chip to recreate the renal microenvironment while allowing for fluid flow [31] (Figure 2C). Employing this system, these researchers identified the mechanosensitive proteins RAC1 and FOS as potential therapeutic targets, underscoring the limitations of traditional models in replicating disease pathology. Organoid-on-a-chip systems can also integrate biosensors and make use of external stimuli to assess real-time response mechanisms and monitor microenvironmental parameters, such as pH, oxygen, and metabolite levels [7,13,40]. Highly integrated microfluidic sensing modules enable highly sensitive and automated online monitoring of organoid behavior [41]. As a result, researchers can analyze the self-assembly of organoids and the changes occurring during disease development at high spatial and temporal resolution [42]. For example, neurodegenerative disease conditions are often associated with neurodevelopmental processes, so the midbrain organoid chip model with integrated optical, electrical, and electrochemical sensors developed by Spitz et al. enables non-invasive multi-parameter monitoring of oxygen and dopamine as well as analysis of electrophysiological activity based on microelectrode arrays, thereby facilitating the investigation of the pathogenesis of Parkinson’s disease, particularly the degenerative process dopaminergic neurons undergo [43,44]. While examples of the use of organoids-on-chips technology for the investigation of rare diseases are currently limited, the extremely customizable and flexible nature of this platform makes it ideally suited for exploring the pathogenesis of and therapeutic options for rare diseases.

Drug development is essential to the study and management of rare diseases. Rare mutations in patients with rare diseases can manifest in complex clinical symptoms, and drug development requires personalized and precise preclinical evaluation strategies that are costly for most patients. On average, the process of developing a new drug requires 10 years and anywhere from USD 500 million to USD 1 billion, necessitating a high level of investment for a relatively low output. This contributes to a lack of affordable or accessible treatment options for rare diseases. The gene-therapy-based treatment of hemophilia B, for instance, costs upwards of USD 3.5 million per case [2]. The use of organoids-on-chips technology has the potential to accelerate the timeline of drug development while limiting associated costs. As organoid-on-a-chip platforms combine the dual advantages of miniaturization and higher throughput, they readily enable drug screening, development, and sensitivity-testing efforts [33]. High-throughput microwell arrays have already been successfully employed to reduce detection timing and costs. In one such example, Hu et al. produced a microwell chip capable of batch-culturing organoids from lung cancer patients, enabling researchers to predict personalized drug responses in just 1 week with a high degree of concordance with actual clinical outcomes [45]. Effectively detecting drug efficacy and toxicity is vital to the development of new therapies for rare diseases. Individualized organoid-on-a-chip systems that reflect patient-specific genetic and pathological features can enable researchers to conduct targeted Phase I clinical trials with superior efficacy and minimal toxicity [26]. For instance, Cui et al. designed a reproducible, high-throughput drug-screening system for colorectal cancer exhibiting low variability suitable for representing the heterogeneity of actual patient treatment responses in a manner closely correlated with clinical outcomes [46]. Notably, organoid-on-a-chip systems can be leveraged to predict the systemic pharmacokinetic properties of drug candidates by integrating different organ chips (e.g., liver, kidney, and heart). Qin et al. used such an approach to generate a liver–islet interaction model mimicking the pathology of type 2 diabetes and allowing them to test the therapeutic effects of metformin, confirming its ability to alleviate glucose-induced pathology [47]. These multi-organ systems are capable of modeling Marfan syndrome and other complex, systemic rare diseases, offering a means of rapidly assessing the pharmacodynamic properties of drug candidates to aid in the treatment of rare diseases.

Organoid transplantation may also be a viable approach to treating certain rare diseases. Despite the therapeutic promise of fetal cells, their attendant ethical concerns and scarcity have impeded their widespread use. As genetic factors are frequently drivers of rare diseases, biomimetic organoids can replace the tissues that are dysfunctional as a consequence of these genetic mutations [48]. Kopan et al., for instance, successfully transplanted patient-derived iPSC-based kidney organoids under the renal capsule in immunodeficient mice and found that delayed angiogenic processes during development contributed to the development of a rare autosomal recessive form of renal tubular insufficiency [49]. The rare gynecological disorder Asherman syndrome (AS) is characterized by severe uterine fibrosis and adhesions that damage the basal lamina and cause infertility. Using a mouse model of AS, Hwang et al. found that endometrial organoid transplantation was sufficient to restore mitochondrial functionality, alleviate fibrosis, normalize the metabolic conditions in the uterus, and improve fertility [50]. These studies have demonstrated that organoid transplantation, as a promising regenerative therapy, contributes to the elucidation of the mechanisms underlying the pathogenesis of rare diseases. However, organoid transplantation can often fail as a consequence of immune-mediated rejection. To address this issue, researchers have explored the use of biocompatible microencapsulation strategies that help isolate these cells such that they can evade immunological clearance following their transplantation. Zhao et al., for instance, used a microfluidic electrospray approach to produce microcapsule-enclosed hiPSC-derived brain organoids. The injection of these organoids into damaged neural tissues resulted in neural regeneration and enhanced functional connectivity among neurons. This strategy may thus provide a new opportunity for the management of rare neurological diseases. The integration of organoid technology with organ transplantation is still only in its infancy [51], and through organoid chip technology, this technique may change the paradigm of transplantation and organoid research and provide new strategies for the treatment of rare diseases.

## 5. Conclusions and Perspectives

The synergistic innovations of organ-chip and organoid technologies have opened up a revolutionary path for rare-disease research. By accurately reproducing the mechanical environment, multicellular types, and biochemical gradients of organ development, we can mimic the pathological characteristics of rare-disease-affected organs. By using cells from patients with rare diseases, organoids-on-chips technology allows for the personalization of therapeutic regimens, together with the refinement of dosing, the optimization of drug efficacy, and the minimization of toxicity, thereby reflecting clinical diversity and aiding clinical trial design for rare diseases. Microchips with microporous structures or microarrays can facilitate the high-throughput culturing of organoids in the context of dynamic, multi-gradient drug screening, thereby supporting the development of rare-disease treatments. Certain systemic rare diseases, including Marfan syndrome and mitochondrial disorders, are characterized by complex interactions among organ systems that organoid-on-a-chip systems are better suited to modeling, allowing researchers to more reliably study drug efficacy and toxicity in affected patients. By capturing the inherent diversity of clinical data, research conducted using organoids-on-chips technology may provide a means with which to improve the success rates of clinical trials aimed at addressing rare diseases.

Despite the striking advances made in recent years, many barriers to the construction of 3D organoid chip models that accurately mirror the pathophysiology of human rare diseases remain. Notably, current organoid-on-chip models lack functional vascular networks, relying on passive diffusion for nutrient supply, which limits long-term culture stability and metabolic support for complex tissue maturation. It also limits the construction of models for oxygen-dependent diseases (e.g., mitochondrial diseases) and restricts studies that translate short-term drug responses into chronic disease progression. However, multidisciplinary integration may provide opportunities to address these issues. The use of 3D-bioprinting technology in organoid research enables precise vascular network integration and the construction of more physiologically relevant individualized efficacy prediction models [52,53]. An example in this regard is the integration of two-photon laser direct writing in constructing high-resolution reticulo-vascularized organoids and assemblies to facilitate organoid growth and long-term cultures. In addition, the smallness of the populations of patients with rare diseases underscores the need for their precision treatment based on the integration of data from individual patients. To address the issue of sample scarcity, researchers can construct libraries of organoid biospecimens collected from multiple regions to allow for more effective in vitro analysis and preclinical assessment of rare diseases. High-throughput on-chip screening strategies also have the potential to accelerate the establishment of standardized cell libraries and the development of drugs capable of treating these rare diseases.

Technological convergence is giving rise to a new generation of research paradigms. AI and gene-editing technologies offer further opportunities to advance these goals. AI-based approaches to the optimization of organoid culture conditions, the identification of disease-related markers from morphological data, and the development of new application-specific biomaterials may support rare-disease modeling efforts [54,55]. Gene-editing tools such as CRISPR/Cas9 can introduce or repair specific mutations, enabling organoids to reflect the genetic characteristics of patients with rare diseases and thus closely mimic real pathological states [56]. The use of patient-derived iPSCs to construct disease-like organ libraries, combined with CRISPR/Cas9 gene correction technology, may enable a closed-loop “patient-chip-drug” response validation system, which will not only accelerate the optimization of individualized medication regimens but, more importantly, also establish a quantitative correlation between the in vitro model and the clinical focus. Multi-omics analytical strategies can also help unveil rare-disease-related markers, signaling pathways, and targets for therapeutic intervention. In our opinion, therefore, the truly transformative potential of organoids-on-chips technology to power rare-disease research lies in the fusion of multimodal data. Organoid morphophenotyping will be combined with artificial intelligence, CRISPR-mediated genotype–phenotype mapping, and single-cell genomics to create predictive models of rare-disease progression based on organoid-on-chips models, identifying subtle pathological markers at the onset of a disease and giving hope for the development of new therapies for the disease. In summary, organoid-on-a-chip platforms remain extremely adaptable and promising tools for the modeling and preclinical study of rare diseases. In the coming years, they will likely enable unprecedented drug screening and personalized treatment efforts that may help revolutionize the rare-disease research space.

## 6. Clinical Translation and Ethical Compliance

In addition, organoid chip technology has received attention and gained recognition from regulatory agencies and demonstrated great clinical translational potential in rare-disease research. Drug-regulatory agencies in some countries and regions have begun to consider incorporating organoid chip data into the drug approval process as important additional evidence to support drug safety and efficacy assessments. The use of organoid chip technology for drug discovery programs targeting rare diseases and the screening of multiple compounds with potential therapeutic effects on specific rare diseases will greatly shorten the time required for discovering and developing drugs for treating rare diseases. Notably, the first FDA-approved clinical trial based entirely on organ chip data exemplifies the translational potential of this technology [32]. And as this technology continues to develop and improve, organoid chips will, as per expectations, play a key role in precision medicine for rare diseases, bringing more treatment options and hope to patients.

Organoid-chip-enabled research on rare diseases is making breakthroughs in biomedical and life sciences. But we must also address several regulatory and legal challenges, such as determining how to ensure that this research is ethical and responsible and how to economically constrain and regulate it. Notably, for ethical principles related to reducing animal use, patient-specific tumor organoid or organoid chip models are admirable in rare disease research, but there are still ethical issues regarding their scale-up and clinical translation. Therefore, ethical principles must be strictly adhered to, and patient-derived samples need to be approved by ethical review boards to ensure that such samples are acquired and used ethically [57,58]. In addition, the design of such research should strictly protect patients’ privacy and identity information. From this perspective, we must emphasize that any research on designing human samples must be premised on respecting patients’ rights and interests and following ethical guidelines to ensure the legitimacy and morality of scientific research.

## Figures and Tables

**Table 1:** Summary of organoids-on-chips technology in rare-disease research.

Category	Key Information
Research background	1. Rare diseases: Over 7000 rare diseases affect ~560 million people globally, with 80% being hereditary and 50% of them emerging in childhood. 2. Unmet needs: Only ~700 drugs (addressing 6% of rare diseases) have been approved. A total of 94% of rare diseases lack effective treatments. 3. Model flaws: Traditional models (2D cell cultures and animal models) fail to recapitulate human-specific pathologies, genetic heterogeneity, and complex microenvironments, leading to a high quantity of drug trial failures.
Core technologies	1. **Organoids:** Organoids have self-assembled 3D structures and are used in organ development studies and the modeling of human physiopathology and drug responses. They also exhibit regenerative potential and allow patient origin models to be constructed. 2. **Organ chips:** These chips simulate in vivo microenvironment mechanics (shear stress, stretch, etc.), biochemistry (oxygen gradients, extracellular matrix, etc.), etc., and interact with multiple organs through microfluidic systems. Biomaterials and biosensors can easily be integrated into the chips.
Combined advantages	**Organoids-on-chips technology:** 1. Controlled integration of multiple microenvironmental factors recapitulates genetic heterogeneity (e.g., monogenic disorders, chromosomal abnormalities, etc.) and the complex pathologic phenotypes of rare diseases. 2. This multimodal research platform enables disease modeling, drug screening, real-time monitoring (using integrated sensors to detect pH, metabolite changes, etc.), and the simulation of multi-organ interactions.
Applications in rare diseases	1. Disease modeling: Recapitulates development defects; captures intra-disease heterogeneity. 2. Drug development: Can be used in high-throughput screening and multi-organ toxicity/pharmacokinetics modeling. 3. Transplantation therapy: Biomimetic organoids can be used to replace dysfunctional tissues; microencapsulation strategies can be used to mitigate immune rejection.
